# A pyroptosis-related lncRNA risk model for the prediction of prognosis and immunotherapy response in head and neck squamous cell carcinoma

**DOI:** 10.3389/fonc.2024.1478895

**Published:** 2024-11-12

**Authors:** Jingyuan Ren, Bingrui Yan, Xurui Wang, Yifei Wang, Qiuying Li, Yanan Sun

**Affiliations:** ^1^ Department of Otorhinolaryngology, Head and Neck Surgery, The Second Affiliated Hospital, Harbin Medical University, Harbin, China; ^2^ Department of Head and Neck Surgery, Jilin Cancer Hospital, Changchun, China; ^3^ Department of Pathology, Jilin Cancer Hospital, Changchun, China

**Keywords:** head and neck squamous cell carcinoma, pyroptosis, lncRNA risk score model, overall survival, immunotherapy response

## Abstract

**Background:**

Recent research has highlighted pyroptosis as a key factor in cancer progression. This study aims to explore the association between pyroptosis-related signatures and overall survival (OS) in head and neck squamous cell carcinoma (HNSC) and develop a pyroptosis-related long non-coding RNA (lncRNA) risk model to predict prognosis and response to immunotherapy in HNSC.

**Methods:**

We extracted expression data for 18 pyroptosis-related genes and identified lncRNA probes specific to HNSC by using datasets from the Gene Expression Omnibus (GEO) and The Cancer Genome Atlas (TCGA). Consensus clustering was performed to categorize HNSC patients into distinct subtypes. A six-lncRNA risk score model was constructed through univariate and least absolute shrinkage and selection operator (LASSO) Cox regression analyses. We evaluated the predictive ability of the lncRNA model for patients’ survival and immunotherapy response. Gene expression was evaluated using immunohistochemistry (IHC) and Reverse Transcription Quantitative Polymerase Chain Reaction (RT-qPCR).

**Results:**

Our analysis revealed two distinct pyroptosis-related subtypes in HNSC patients, Cluster A and Cluster B. Notably, patients in Cluster B exhibited significantly poorer overall survival compared to those in Cluster A. Through differential expression analysis, we identified six lncRNAs (AC002331.1, CTA-384D8.35, RP11-291B21.2, AC006262.5, RP1-27K12.2, and RP11-54H7.4) that were differentially expressed between these clusters. A 6-lncRNA risk score model was developed, which successfully stratified patients into high- and low-risk groups with distinct overall survival outcomes. Validation using RT-qPCR confirmed the differential expression of these six lncRNAs in HNSC tumor tissues compared to adjacent normal tissues, we found that the expression of CTA-384D8.35 was significantly increased in the tumor group (t=-6.203, P<0.001). Furthermore, the 6-lncRNA risk score model demonstrated a significant association with patient response to immunotherapy, with the low-risk group exhibiting a higher objective response rate to immune checkpoint blockade (ICB) therapy and longer survival compared to the high-risk group.

**Conclusion:**

Our study underscores the role of pyroptosis signatures in HNSC prognosis and identifies two distinct pyroptosis subtypes with differing survival outcomes. The six-lncRNA risk score model offers a valuable tool for predicting patient prognosis and potential benefits from ICB therapy. These findings highlight the importance of pyroptosis and associated lncRNAs in the tumor microenvironment, paving the way for novel targeted therapies in HNSC.

## 1 Introduction

Head and neck squamous cell carcinoma (HNSC) is a prevalent and aggressive malignancy, with an estimated annual incidence of around 500,000 cases globally (1). Despite advances in chemotherapy, surgery, and radiation therapy, HNSC remains a formidable challenge, with a mortality rate of approximately 50% (2). Most HNSC cases are diagnosed at advanced stages (III/IV), which are associated with poor prognoses, including a recurrence-free survival rate of about 40% and a five-year overall survival (OS) rate of approximately 60% (2, 3). These statistics highlight the urgent need for novel therapeutic strategies, both single and combinatorial, to improve patient outcomes. Developing new prognostic biomarkers and drug targets is essential for enhancing the clinical efficacy of HNSC treatments.

Pyroptosis is a recently discovered form of programmed cell death. It is marked by cell swelling, protrusion formation, and the development of pores in the cell membrane. These pores disrupt membrane integrity, causing the release of cellular contents and triggering an inflammatory response (4). As pyroptosis progresses, it involves nuclear condensation and DNA fragmentation, culminating in cell death (4, 5). This process involves various inflammatory molecules, including the gasdermin (GSDM) protein family, NOD-like receptors (NLRs), interleukins (ILs), and caspases (6). Caspases, a family of cysteine proteases, play a critical role by degrading intracellular proteins upon activation, leading to cell death. Interleukins facilitate communication between immune cells, influencing cancer development and progression. NOD-like receptors, part of the cytoplasmic pattern recognition receptor family, are crucial for inflammasome formation, which releases IL-1β and IL-18 to induce a pro-inflammatory response (7). Gasdermins execute pyroptosis by forming membrane pores, causing cell swelling and rupture (8, 9).

Pyroptosis is implicated in various diseases, including autoimmune disorders, neurodegenerative conditions, ischemia-reperfusion injuries, and cancer. For instance, resistance to pyroptosis in CD4^+^ T lymphocytes is associated with acute liver and lung injuries (10), while NLRP1 inflammasome-mediated pyroptosis plays a significant role in the neurodegeneration observed in Alzheimer’s disease (11). In the context of cancer, the role of pyroptosis becomes even more intricate. On one hand, pyroptosis can act as a tumor-suppressive mechanism by eliminating transformed cells before they proliferate and form tumors. On the other hand, dysregulation of pyroptosis can contribute to cancer progression and drug resistance. For example, Wang et al. reported that LSD1 silencing inhibits the proliferation, migration, invasion, and epithelial-to-mesenchymal transition of hypopharyngeal cancer cells by inducing autophagy and pyroptosis (12). Indeed, recent studies have linked drug resistance in cancer to pyroptosis and pyroptosis-regulatory genes (PRGs), suggesting that targeting these genes could offer novel therapeutic strategies (13, 14). In HNSC, recent research has underscored the importance of pyroptosis-related gene signatures (15, 16). These signatures have been found to correlate with the tumor microenvironment (TME) and immune cell infiltration, indicating that pyroptosis may play a crucial role in shaping the immune landscape of HNSC tumors (17). By understanding the biological functions and regulatory mechanisms of pyroptosis in this context, researchers can gain insights into the underlying mechanisms of tumor progression and identify potential therapeutic targets.

In recent years, long non-coding RNAs (lncRNAs) have emerged as crucial regulators in various biological processes, including tumorigenesis and cancer progression (18, 19). These lncRNAs, which are non-coding RNAs longer than 200 nucleotides, do not encode proteins but can influence gene expression through mechanisms such as chromatin remodeling, transcriptional regulation, and post-transcriptional processing (20, 21). Aberrant expression of lncRNAs has been linked to numerous cancers, highlighting their potential as biomarkers for cancer diagnosis, prognosis, and therapy. The role of lncRNAs in HNSC has gained increasing attention, with research revealing their involvement in key oncogenic processes like cell proliferation, apoptosis, proptosis, and metastasis (22, 23). For example, LncRNA MEG3 induces renal tubular epithelial cell pyroptosis through the regulation of the miR-18a-3p/GSDMD pathway in lipopolysaccharide-induced acute kidney injury (24). However, the specific role of lncRNAs in pyroptosis—a form of programmed cell death characterized by inflammatory responses—remains unclear in HNSC.

In the present study, we attempt to investigate the role of pyroptosis and its associated lncRNAs in predicting the prognosis of HNSC. Our research innovatively identifies two distinct pyroptosis subtypes with differing survival outcomes and develops a six-lncRNA risk score model that predicts patient prognosis and potential benefits from immune checkpoint blockade (ICB) therapy. By elucidating the biological functions and regulatory mechanisms of pyroptosis in HNSC, our findings offer novel insights into the tumor microenvironment and immune cell infiltration, paving the way for personalized treatment strategies and enhanced clinical outcomes.

## 2 Materials and methods

### 2.1 Patients and specimens

We collected a total of 72 pairs of laryngeal squamous cell carcinoma (LSCC) and adjacent normal tissues from patients who underwent partial or complete laryngectomy at the Department of Otolaryngology, Second Affiliated Hospital of Harbin Medical University, between January 2018 and November 2019. All patients included in the study had not received radiotherapy or chemotherapy prior to tissue collection. The expression levels of pyroptosis-related genes and lncRNAs were assessed using immunohistochemistry (IHC) and reverse transcription quantitative polymerase chain reaction (RT-qPCR). This study was approved by the Ethics Committee of Harbin Medical University, and informed consent was obtained from all participants.

### 2.2 IHC analysis

Patients provided informed consent for participation in the study. The HNSC and adjacent normal tissues were fixed in formalin and embedded in paraffin. Tissue sections (4 μm thick) were deparaffinized, rehydrated, and subjected to antigen retrieval using citrate buffer. After blocking endogenous peroxidase activity with hydrogen peroxide, sections were incubated overnight with primary antibodies specific to various pyroptosis-related genes. Following this, sections were treated with HRP-conjugated secondary antibodies. Immunoreactivity was visualized using DAB, and sections were counterstained with hematoxylin. The stained sections were examined under a light microscope. The protein expression was quantified as average optical density (AOD) using Image J software.

### 2.3 Public databases

To obtain and analyze public gene expression data and comprehensive clinical annotations, we accessed datasets from the Gene Expression Omnibus (GEO) and The Cancer Genome Atlas (TCGA). RNA sequencing data (FPKM values) and clinical information for TCGA datasets were downloaded from UCSC Xena (https://gdc.xenahubs.net). For the GEO dataset, we retrieved the gene expression matrix file for GSE65858 from the NCBI GEO database (http://www.ncbi.nlm.nih.gov/geo/). Illumina probe sequences were sourced from the GPL10558 annotation file and were uniquely mapped to the human genome (hg38) using NCBI BLAST, ensuring no mismatches. Probes specific to long non-coding RNAs (lncRNAs) were identified by matching probe chromosomal locations with those of lncRNA genes annotated by GENCODE.

Data processing for the TCGA-HNSC and GSE65858 datasets involved the following steps: (1) exclusion of samples lacking clinical follow-up information; (2) exclusion of samples with unknown survival time, survival time of less than 0 days, or missing survival status; (3) conversion of probes to gene symbols; (4) removal of probes mapping to multiple genes; (5) for probes associated with multiple gene symbols, the median expression value was used. The preprocessed TCGA-HNSC dataset included 44 adjacent normal tissue samples and 501 tumor tissue samples, while the GSE65858 dataset comprised 270 tumor tissue samples.

### 2.4 Consensus clustering

To investigate the relationship between the expression patterns of 18 pyroptosis-related genes and patient prognosis, we performed unsupervised and consistent clustering analysis using the TCGA_HNSC cohort. We extracted the expression data for 18 pyroptosis-related genes from the TCGA database. HNSC patients without follow-up information were excluded. The remaining patients were then categorized into distinct subtypes using the “ConsensusClusterPlus” package, with 1000 iterations and an 80% resampling rate. Principal component analysis (PCA) was conducted to evaluate gene expression patterns across the different HNSC subtypes. Survival curves were estimated using the Kaplan-Meier method, and differences in survival between subgroups were analyzed to assess their prognostic significance.

### 2.5 Gene Ontology and Kyoto Encyclopedia of Genes and Genomes pathway analysis

Gene expression data from the TCGA_HNSC cohort were normalized, and differentially expressed genes (DEGs) between pyroptosis subtypes were identified using the limma package in R, with an adjusted P-value < 0.05 and log2(Fold Change) > 0.5. GO enrichment analysis categorized DEGs into Biological Process, Cellular Component, and Molecular Function categories. KEGG pathway enrichment analysis mapped DEGs to pathways in the KEGG database. Both analyses were conducted using the clusterProfiler package in R, and results were visualized with bar plots and dot plots to illustrate the biological significance of DEGs in different pyroptosis subtypes.

### 2.6 Differential expression analysis between distinct subtypes

Based on the expression of pyroptosis-related genes and the results of consensus clustering, tumor samples were divided into two groups: Cluster A and Cluster B. Differential expression analysis between these clusters were performed using the limma package in R. The thresholds for identifying differentially expressed genes were set at a false discovery rate (FDR) < 0.05 and an absolute log2 fold change (|log2(FC)|) > 0.5. Additionally, the expression of lncRNAs was extracted using gene annotation.

### 2.7 Construction of lncRNA risk score model and validation

To develop a robust prognostic signature, we incorporated all pyroptosis-related lncRNAs identified through consensus clustering analysis into a least absolute shrinkage and selection operator (LASSO) Cox regression model. Genes that passed initial screening in univariate analysis were further evaluated using LASSO penalized Cox regression. This method aimed to identify genes significantly associated with HNSC prognosis. The optimal lambda value was determined using the R package “glmnet” through five-fold cross-validation, which minimized the average cross-validation error. Based on this selection process, six key lncRNAs (AC002331.1, CTA-384D8.35, RP11-291B21.2, AC006262.5, RP1-27K12.2, and RP11-54H7.4) and their coefficients were used to construct the prognostic model. The risk score formula was structured as follows:


Risk_scores=∑Coef(i)*Exp(i)

where Cofi was the coefficient, Exp(i) was the expression of screened lncRNAs. This formula was used to calculate the risk score of all patients in our study. The final 6-lncRNA gene signature formula is: RiskScore = (-0.081)*AC002331.1 + (0.056)*AC006262.5 + (-0.074)*CTA.384D8.35 + (-078)*RP11.291B21.2 + (0.065)*RP11.54H7.4 + (0.149)*RP1.27K12.2.

### 2.8 Kaplan-Meier survival analysis and model validation

Kaplan-Meier survival analysis was conducted using the “survival” and “survminer” R packages to compare overall survival (OS) between low-risk and high-risk groups. The “survival ROC” R package was utilized for time-dependent ROC curve analysis to evaluate the predictive accuracy of the prognostic signature. Additionally, univariate and multivariate Cox regression analyses were performed to assess the prognostic value of the signature relative to clinical features.

### 2.9 Immune status and immunotherapy response analysis

We compared tumor-infiltrating immune cell proportions between high-risk and low-risk groups using several algorithms, including CIBERSORT, CIBERSORT-ABS, QUANTISEQ, XCELL, MCPcounter, EPIC, and TIMER. The “GSVA” package facilitated Single Sample Gene Set Enrichment Analysis (ssGSEA) to evaluate immune function and pyroptosis levels in both risk groups. Differences in the expression of immune checkpoint molecules between the risk groups were assessed using the Wilcoxon test. To investigate the prognostic value of the signature in the context of immunotherapy, ssGSEA was performed on four cohorts treated with anti-PD-L1/PD-1/CTLA-4 therapies. Patients were categorized into high and low pyroptosis score groups based on the median pyroptosis score, and survival analyses were conducted.

### 2.10 Functional enrichment analysis

Gene Set Enrichment Analysis (GSEA) was carried out to examine Kyoto Encyclopedia of Genes and Genomes (KEGG) pathways between the high-risk and low-risk groups based on the pyroptosis-related lncRNA signature. This analysis was performed using GSEA software (version 4.1.0).

### 2.11 The correlations between the expression of lncRNAs and pyroptosis-related genes

In our study, we obtained gene expression data for head and neck squamous cell carcinoma (HNSC) from the TCGA database and preprocessed the data by removing genes with zero expression across all samples, followed by normalization and log2 transformation of the remaining gene expression values. Subsequently, we calculated the Spearman correlation coefficients and their p-values between the expression of specific lncRNAs and a list of predefined pyroptosis-related genes.

### 2.12 RT-qPCR

Total RNA was extracted from HNSC tumor and adjacent normal tissues using TRIzol reagent. RNA purity and concentration were assessed to ensure quality. For cDNA synthesis, 1 μg of RNA was reverse transcribed using a PrimeScript RT reagent kit. qPCR was conducted with SYBR Green Master Mix on a QuantStudio 3 Real-Time PCR System, using specific primers for selected lncRNAs. The PCR protocol included an initial denaturation step followed by 40 cycles of denaturation and annealing/extension. Relative expression levels of lncRNAs were determined using the 2^−ΔΔCt method, with GAPDH serving as the internal control. The primers used in this study are listed in **Supplementary Table S1**.

### 2.13 Statistical analysis

Statistical analyses were performed using R software (Version 4.1.0) and SPSS (Version 23.0). The Chi-squared test was employed to examine the relationship between the prognostic signature and clinicopathological features. Univariate and multivariate Cox regression analyses were conducted to identify independent factors associated with OS prognosis. ROC analysis was used to evaluate the predictive accuracy of the prognostic model. Statistical significance was defined as P < 0.05.

## 3 Results

### 3.1 Expression and survival correlation of pyroptosis-related genes in HNSC

**Figure 1:**
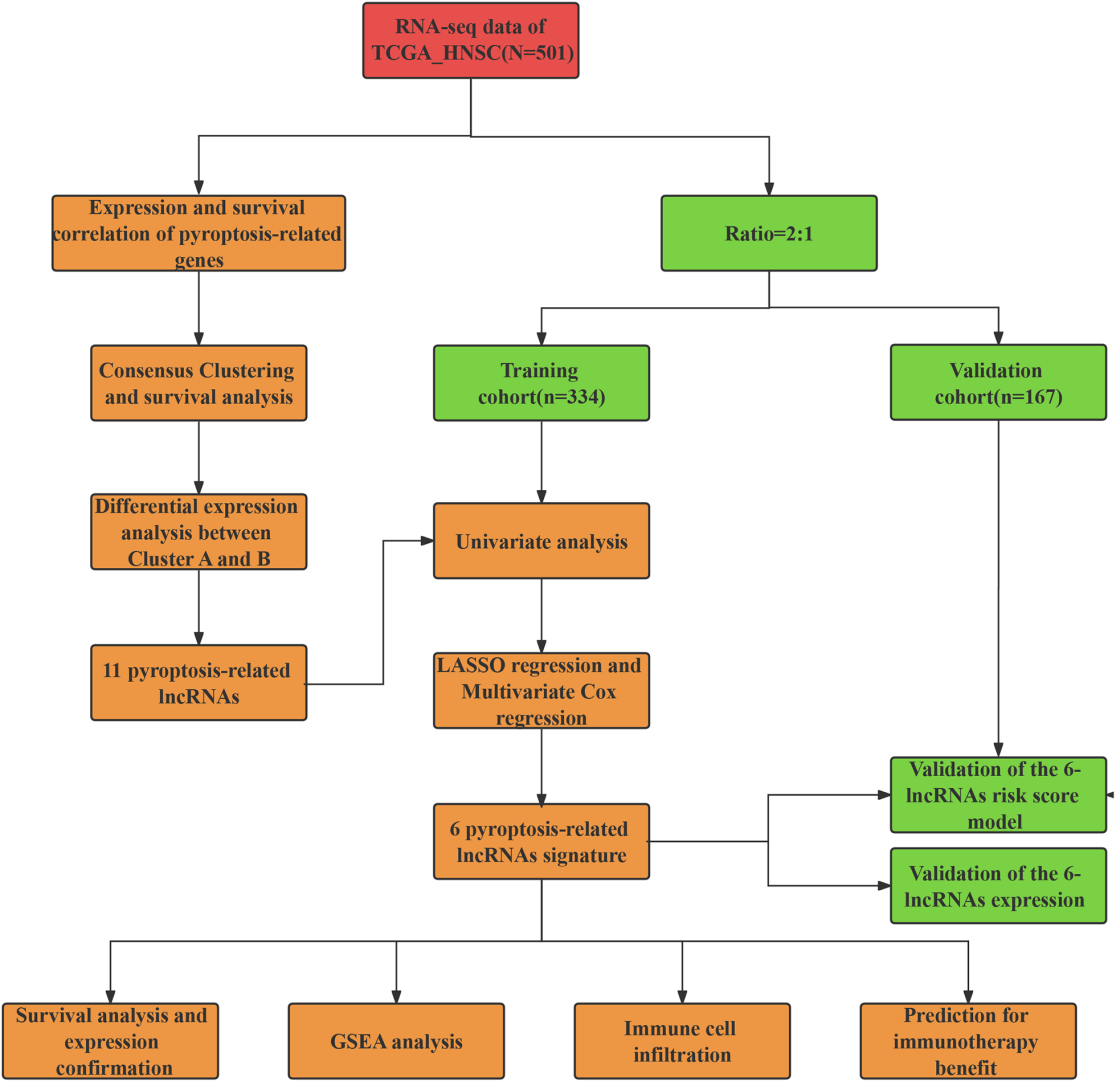
The flow chart of our study.

**Figure 2:**
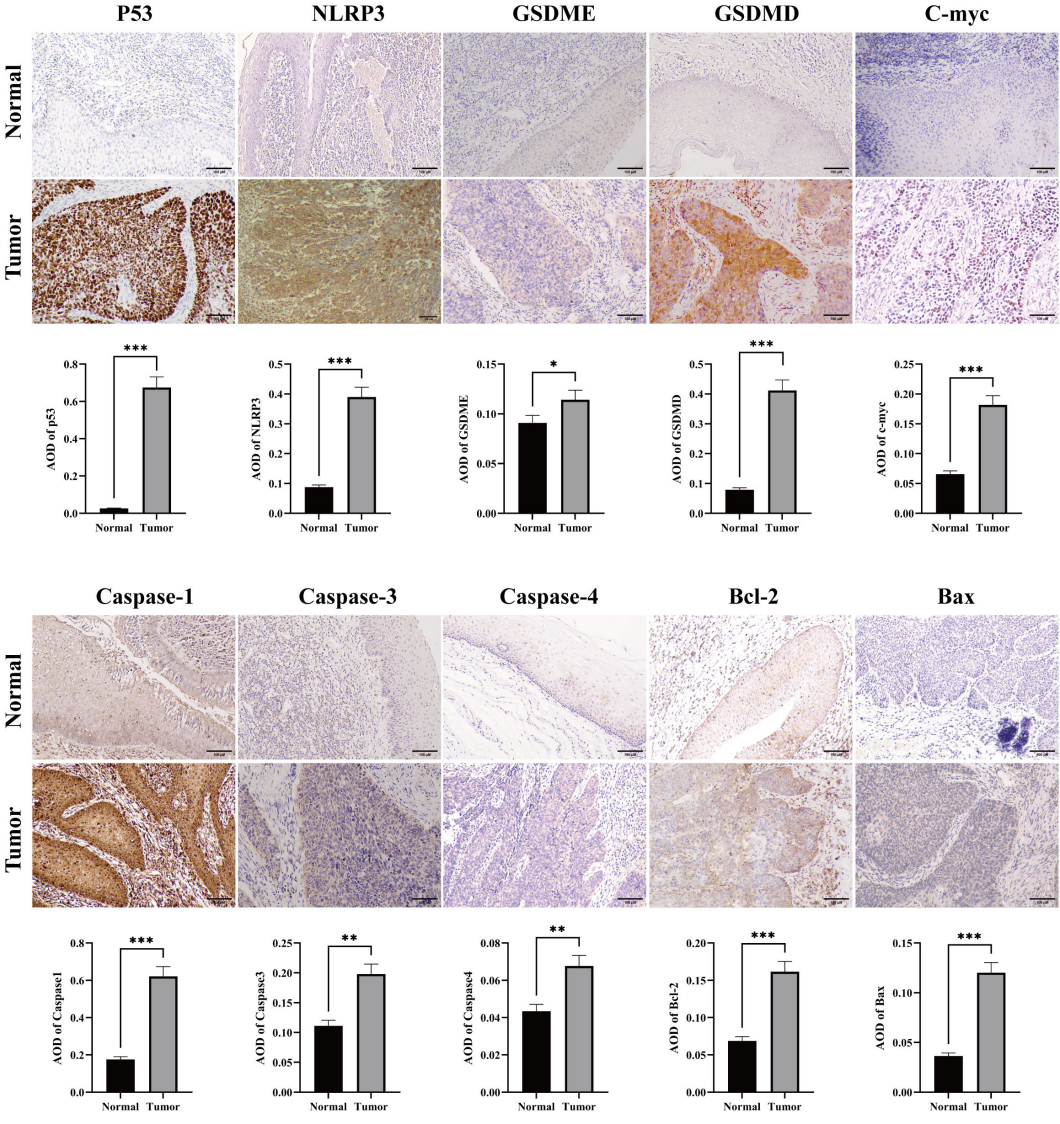
Immunohistochemical staining for apoptosis and pyrolysis-related gene expression in HNSC tumor and normal tissues. Representative images showed the expression levels of P53, NLRP3, GSDME, GSDMD, C-myc, Caspase 1, Caspase 3, Caspase 4, Bcl-2 and Bax in HNSC tumor tissues compared to adjacent normal tissues. Scale bar = 100 μm. *p<0.05, **p<0.01, ***p<0.001.

The workflow of our study is illustrated in **Figure 1**. We first conducted a histological evaluation of apoptosis and pyroptosis-related genes in HNSC. As shown in **Figure 2**, the expression levels of P53, NLRP3, GSDME, GSDMD, C-myc, Caspase-1, Caspase-3, Caspase-4, Bcl-2 and Bax were significantly upregulated in HNSC tumor tissues compared to normal tissues.

**Figure 3:**
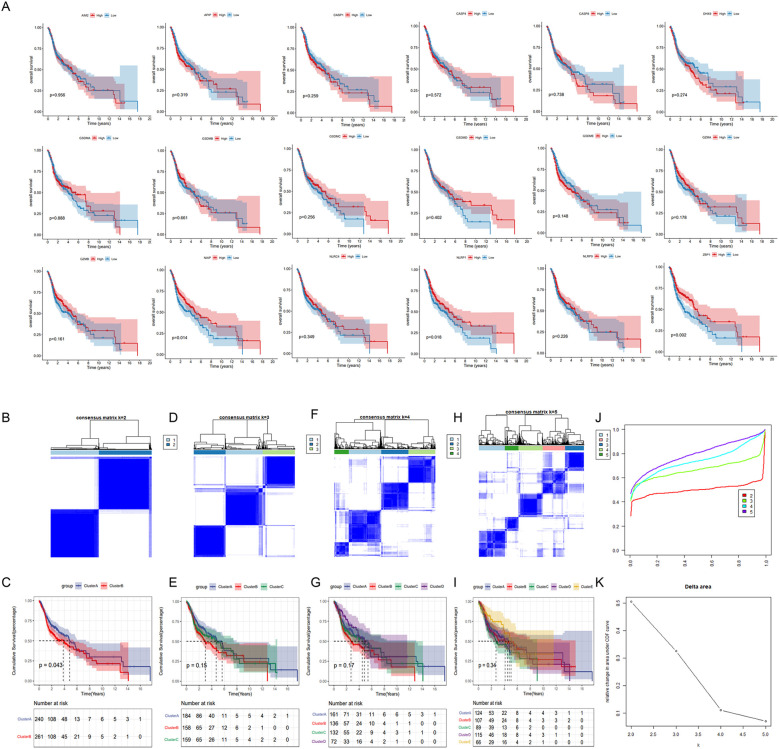
Identification of pyroptosis subtypes in HNSC patients. **(A)** The data were obtained from TCGA_HNSC data set. Patients were divided into high and low expression groups for each of the 18 pyrolysis-related genes based on median expression levels. Kaplan-Meier survival curves were generated to assess the correlation between gene expression and overall survival. **(B, D, F, H)** Unsupervised consensus clustering analysis of the TCGA_HNSC cohort using 18 pyroptosis-related genes with clustering variable (κ) ranging from 2 to 5. **(C, E, G, I)** Kaplan-Meier survival curves comparing overall survival between distinct pyroptosis subtypes. **(J)** Consensus clustering cumulative distribution function (CDF). **(K)** The relative variation of the area under CDF curve.

We identified eighteen pyroptosis-related genes in each sample of the TCGA_HNSC dataset. Patients with HNSC were divided into high-expression and low-expression groups for each gene, based on median expression levels. The correlation between pyroptosis-related genes and overall survival (OS) was depicted in **Figure 3A**. Most pyroptosis-related genes did not show a significant correlation with OS. Notable exceptions included NAIP, NLRP1, and ZBP1, where high expression levels were significantly associated with improved OS.

### 3.2 Identification of pyroptosis subtypes and differentially expressed genes in HNSC

Distinct pyroptosis subtypes was identified using a consistent clustering analysis. As shown in **Figure 3**. The clustering variable (k) was explored within the range of 2 to 5. The results indicated that the intra-group correlation was highest at k = 2, with low inter-group correlation, which allowed for a relatively stable separation of samples in the TCGA dataset (**Figures 3B–K**). Furthermore, significant differences in overall survival (OS) were observed only at k = 2, suggesting that HNSC patients could be divided into two distinct pyroptosis subtypes: Cluster A and Cluster B. Cluster A exhibited a median survival time of 4.9 years, demonstrating a better prognosis compared to Cluster B, which had a median survival time of 3.9 years (**Figure 3B**).

**Figure 4:**
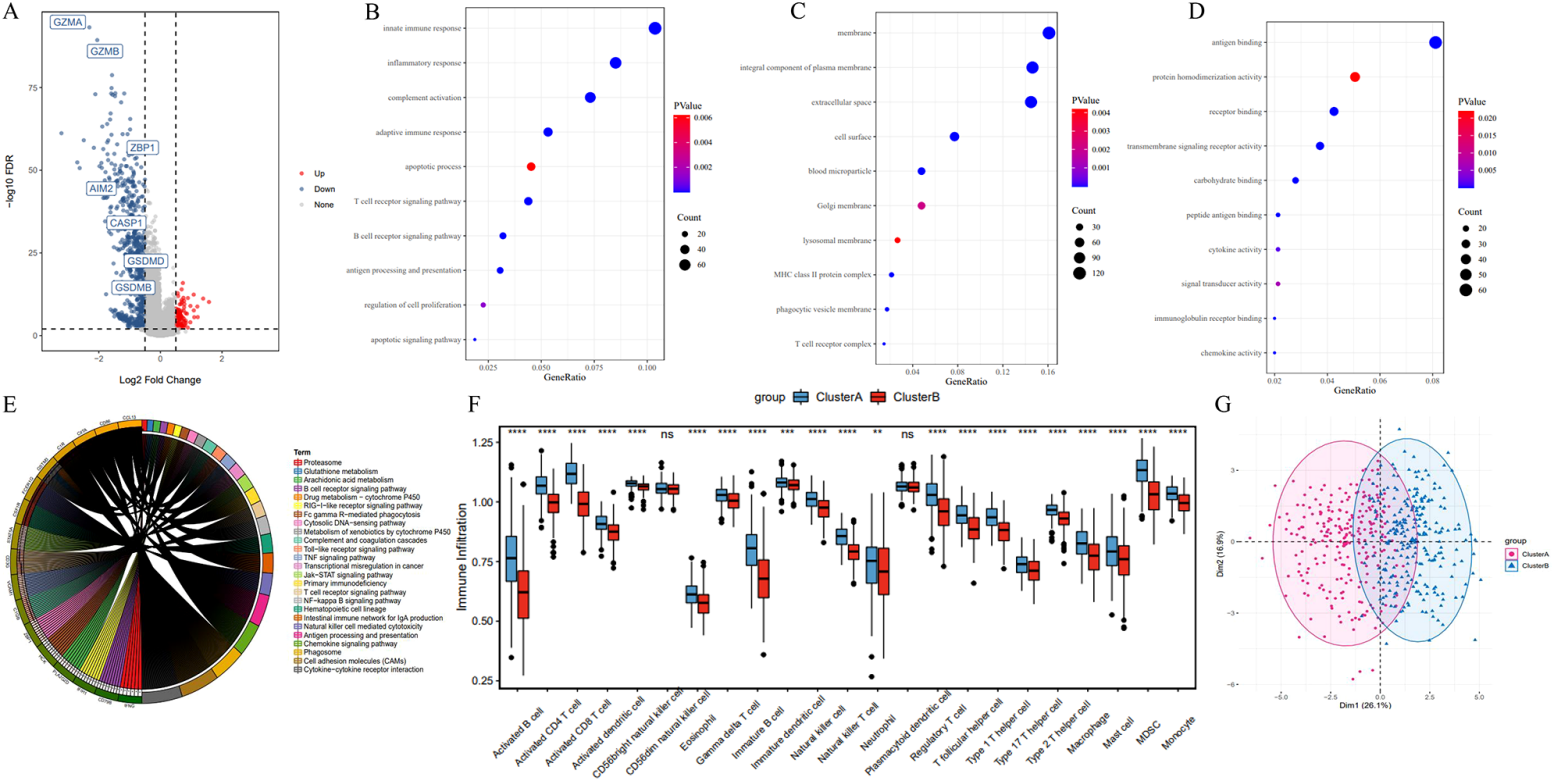
Differentially expressed genes and functional enrichment analysis between pyroptosis subtypes. **(A)** Volcano plot of differentially expressed genes between Cluster A and Cluster B. **(B–D)** Gene Ontology (GO) enrichment analysis of differentially expressed genes, highlighting enrichment in cellular components (e.g., plasma membrane), molecular functions (e.g., antigen binding), and biological processes (e.g., immune response). **(E)** KEGG pathway enrichment analysis. **(F)** ssGSEA results indicating reduced immune cell infiltration in Cluster B compared to Cluster A, including activated B cells and T cells. **(G)** PCA analysis of expression profiles in Cluster A and B. *P<0.05, **P<0.01 and ***P<0.001.

Next, we performed a differential gene expression analysis between cluster A and B. Among 779 differentially expressed genes, 107 genes were highly expressed in Cluster B, while 675 genes were highly expressed in Cluster A (**Figure 4A**). GO functional enrichment analysis revealed that these differentially expressed genes were primarily associated with functions related to cell surface, plasma membrane, lysosomal membrane, phagocytic vesicle membrane, antigen binding, receptor binding, immunoglobulin receptor binding, and peptide antigen binding. These genes were involved in biological processes such as immune response, inflammatory response, apoptosis, T cell receptor signaling, B cell receptor signaling, antigen processing and presentation, and cell proliferation (**Figures 4B–D**).

KEGG enrichment analysis showed that differentially expressed genes in the two subtypes were predominantly enriched in immune-related pathways and tumorigenesis-promoting pathways, including antigen processing and presentation, cell adhesion molecules, phagosome, primary immunodeficiency, chemokine signaling, tumor necrosis factor signaling, Jak-STAT signaling, and Toll-like receptor signaling pathways (**Figure 4E**). Additionally, Single Sample Gene Set Enrichment Analysis (ssGSEA) revealed a significantly reduced degree of immune cell infiltration in Cluster B, including Activated B cells, Activated CD8^+^ T cells, Activated CD4^+^ T cells, Natural Killer cells, and Macrophages (**Figure 4F**). In the PCA plot, red points and blue points were separated, which indicated that cluster A, cluster B could be distinguished based on the expression of pyroptosis-related genes (**Figure 4G**). These findings suggest that the poorer prognosis associated with the Cluster B pyroptosis subtype may be related to decreased immune cell infiltration.

### 3.3 Construction of the 6-lncRNA risk score model

lncRNAs are increasingly recognized for their potential in predicting tumor prognosis (25–27). In this study, we developed a risk score model for HNSC based on differentially expressed lncRNAs (n=11) identified between the two pyroptosis subtypes. The correlations between the expression of 11 lncRNAs and pyroptosis-related genes were showed in **Supplementary Figure S1**. The results suggested that the expression of each lncRNA was significantly associated with most of pyroptosis-related genes. The TCGA_HNSC dataset (n=501) was split into a training set (n=334) and a test set (n=167) in a roughly 2:1 ratio.

**Figure 5:**
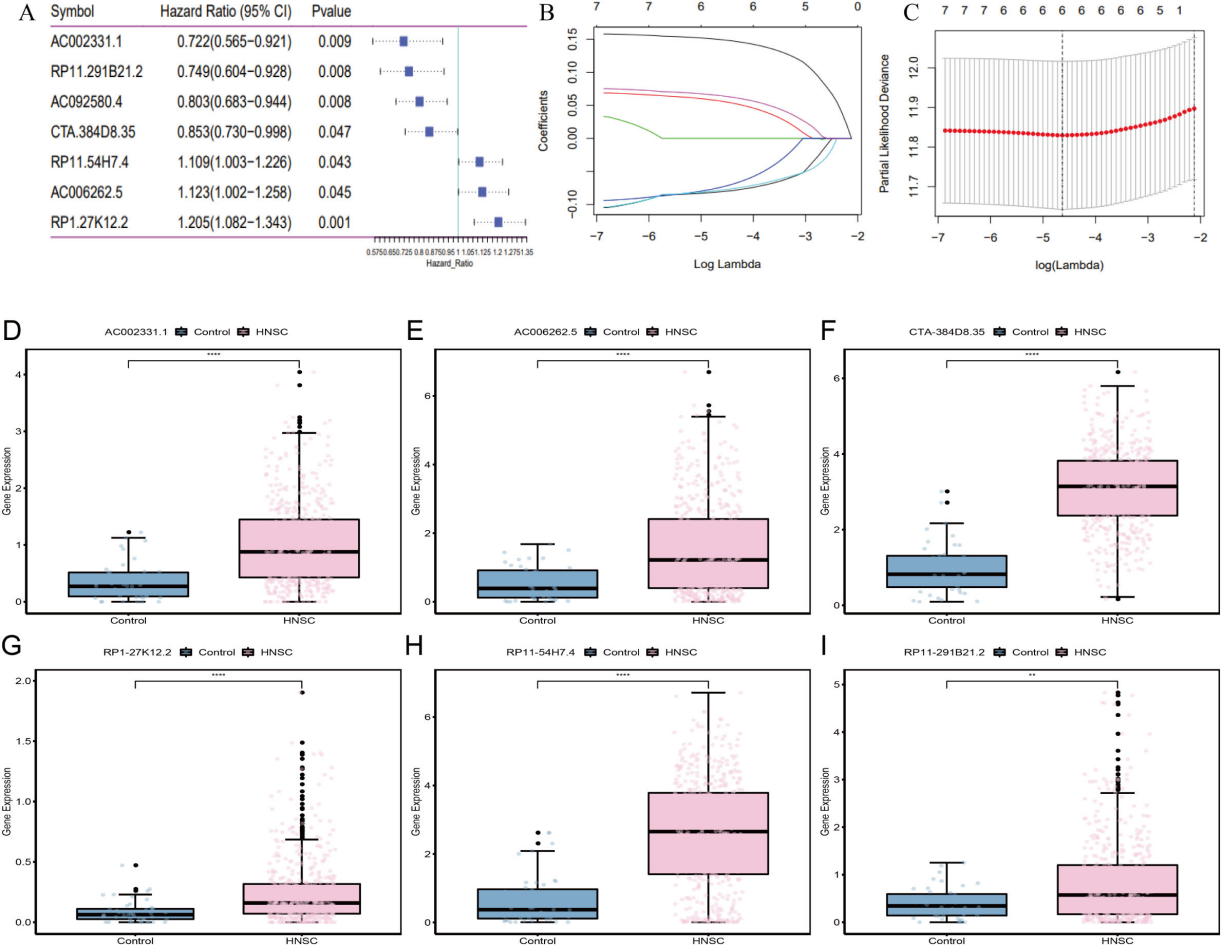
Construction of the 6-lncRNA risk score model in HNSC. **(A)** Univariate COX analysis. **(B)** The change track of each independent variable. The horizontal axis represents the log value of lambda, and the vertical axis represents the coefficient of the independent variable. **(C)** Confidence intervals under each lambda. **(D–I)** Expression levels of the 6 lncRNAs in tumor versus adjacent normal tissues from TCGA_HNSC cohort.

In the training set, univariate Cox regression analysis identified 11 candidate lncRNAs with a significance threshold set at p < 0.05, resulting in the selection of 7 lncRNAs (**Figure 5A**). Subsequently, LASSO regression was employed to further refine the variables, ultimately retaining 6 lncRNAs: AC002331.1, CTA-384D8.35, RP11-291B21.2, AC006262.5, RP1-27K12.2, and RP11-54H7.4 (**Figures 5B, C**).

**Figure 6:**
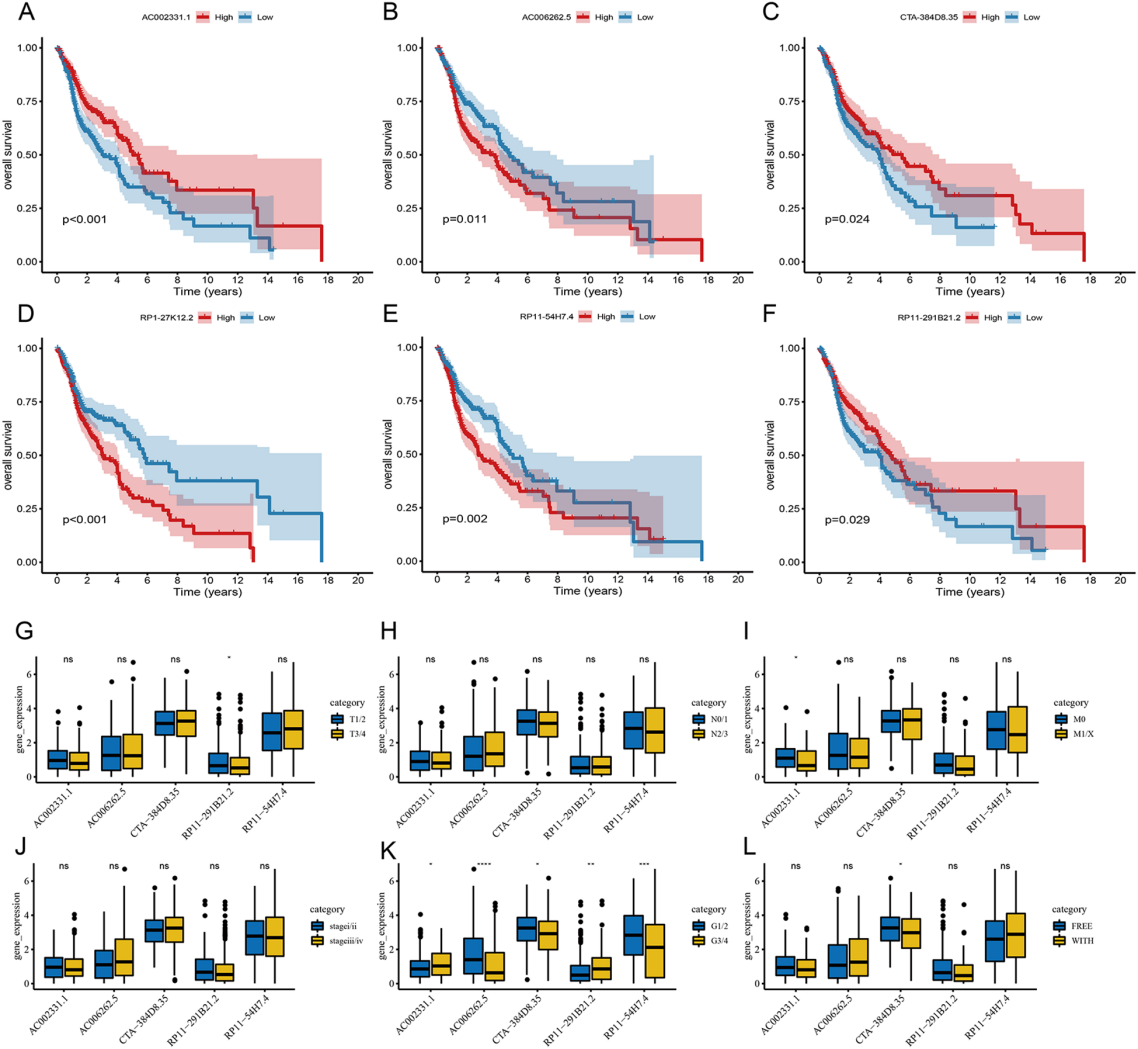
Prognostic significance of the 6-lncRNA risk score model in HNSC. **(A–F)** Kaplan-Meier survival analysis for the correlation of AC002331.1, CTA-384D8.35, RP11-291B21.2, AC006262.5, RP1-27K12.2 and RP11-54H7.4 with the overall survival of patients with HNSC. **(C)** The correlation of AC002331.1, CTA-384D8.35, RP11-291B21.2, AC006262.5, and RP11-54H7.4 with patients’ clinical characteristics. *P<0.05, **P<0.01 and ***P<0.001. ns refers to the absence of significant statistical significance..

To evaluate the roles of these 6 lncRNAs in HNSC, the Wilcoxon test was used to compare their expression levels between tumor and adjacent normal tissues. All 6 lncRNAs were significantly upregulated in tumor tissues (**Figures 5D–I**). Tumor samples were categorized into high and low expression groups based on the median expression levels. Kaplan-Meier survival analysis revealed that high expression of AC002331.1, CTA-384D8.35, and RP11-291B21.2, as well as low expression of AC006262.5, RP1-27K12.2, and RP11-54H7.4, were associated with improved patient prognosis (**Figures 6A–F**).

Furthermore, we examined the expression of these 6 lncRNAs across various clinical features. RP11-291B21.2 was found to be more highly expressed in T1/2 stages compared to T3/4 stages, and AC002331.1 showed higher expression in M0 compared to M1/X stages. Additionally, all 6 lncRNAs displayed varying levels of differential expression in cancer grading (**Figures 6G–L**).

**Figure 7:**
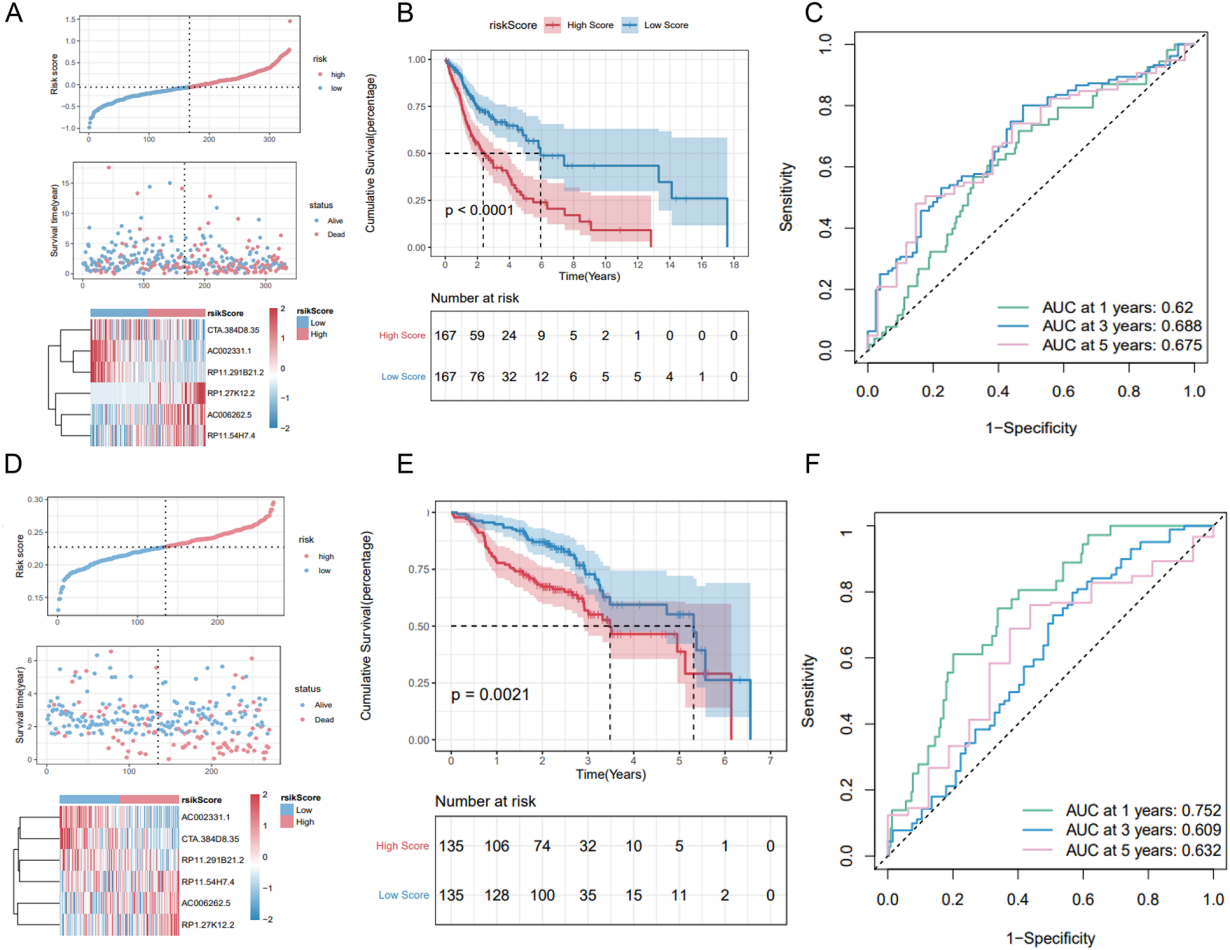
Validation of the 6-lncRNA risk score model. **(A)** Proportion of death samples in high and low-risk groups in the training set. **(B)** Kaplan-Meier survival curves in the training set showing poorer survival in the high-risk group. **(C)** ROC curves for the training set with 1-, 3-, and 5-year AUCs of 0.625, 0.610, and 0.671, respectively. **(D–F)** Risk score distribution, survival status and ROC curves of patients in GSE65858 dataset.

The Riskscore of each sample was calculated. In the training set, samples were classified into high-risk and low-risk groups based on the median risk score. The high-risk group exhibited a higher proportion of deaths (**Figure 7A**). Kaplan-Meier analysis revealed that patients with high risk scores had significantly poorer OS compared to those with low risk scores (**Figure 7B**). The model demonstrated robust predictive power for OS, with 1-, 3-, and 5-year AUCs of 0.620, 0.688, and 0.675, respectively (**Figure 7C**).

### 3.4 Validation of the 6-lncRNA risk score model

We validated the predictive capability of the risk score model using the TCGA_HNSC test set and the overall TCGA_HNSC dataset. Risk scores for each sample in the test set were calculated using the same formula. Samples were then categorized into high-risk and low-risk groups based on the median risk score. As shown in **Supplementary Figure S2A**, the high-risk group had a higher proportion of death cases. Kaplan-Meier analysis demonstrated significantly lower overall survival in the high-risk group compared to the low-risk group (**Supplementary Figure S2B**).

In the TCGA_HNSC test set, the risk score model exhibited good predictive performance for OS, with 1-, 3-, and 5-year AUCs of 0.625, 0.610, and 0.671, respectively (**Figure S2C**). Similarly, in the overall TCGA_HNSC dataset, the model demonstrated strong predictive capability with 1-, 3-, and 5-year AUCs of 0.622, 0.660, and 0.675, respectively (**Supplementary Figures S1D–F**).

To further evaluate the stability of the risk score model, we analyzed the GSE65858 dataset from the GEO database. Applying the same formula, we calculated risk scores and classified samples into high-risk and low-risk groups. Consistent with our previous findings, the high-risk group exhibited a higher proportion of deaths (**Figure 7D**) and significantly poorer overall survival (OS) (**Figure 7E**). The 1-, 3-, and 5-year AUCs for the GSE65858 dataset were 0.752, 0.609, and 0.632, respectively (**Figure 7F**).

In summary, these results validate the robustness of the 6-lncRNA risk score model in predicting overall survival across various datasets.

### 3.5 Relationship between tumor risk score and immune cell infiltration

**Figure 8:**
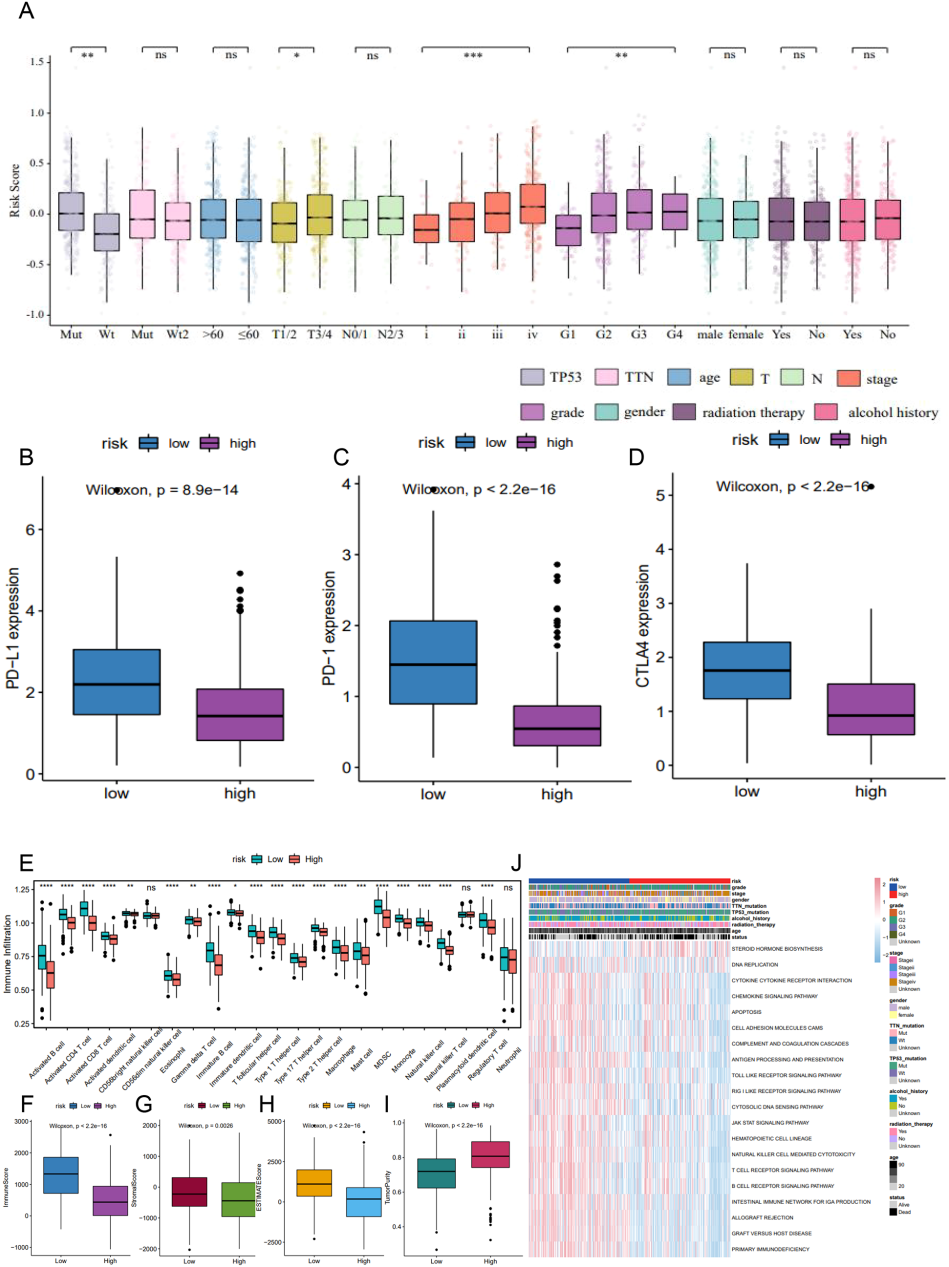
The correlation of 6-lncRNA risk score with immune cell infiltration in HNSC. **(A)** Differences in risk scores across clinicopathological characteristics including TP53 mutation, T staging, tumor stage, and tumor grade. **(B–D)** Comparison of immune checkpoint expression (PD-L1, PD-1, CTLA4) between high and low-risk groups. **(E)** ssGSEA analysis for immune cell infiltration in the high and low-risk groups. **(F–I)** ESTIMATE algorithm analysis showing the immune, stromal, and ESTIMATE scores, and tumor purity in the high and low-risk groups. **(J)** GSVA analysis indicating immune-related pathways and active metabolic pathways in the high and low-risk groups. *P<0.05, **P<0.01 and ***P<0.001. ns refers to the absence of significant statistical significance.

We next examined the prognostic relevance of the 6-lncRNA risk score model in relation to various clinicopathological characteristics of HNSC patients. Significant differences in risk scores were observed across TP53 mutation status, T staging, tumor stage, and tumor grade (**Figure 8A**). Additionally, high-risk patients displayed lower expression levels of immune checkpoint molecules (PD-L1, PD-1, and CTLA-4) compared to low-risk patients (**Figures 8B–D**).

Using ssGSEA, we assessed the infiltration of 23 immune cell types in the TCGA_HNSC dataset. The high-risk group exhibited significantly higher infiltration levels of various immune cells, including activated B cells, CD8^+^ T cells, CD4^+^ T cells, eosinophils, regulatory T cells, and monocytes (**Figure 8E**). The ESTIMATE algorithm indicated that high-risk patients had significantly lower immune, stromal, and ESTIMATE scores, alongside higher tumor purity (**Figures 8F–I**). GSVA revealed that metabolic pathways such as DNA replication and steroid hormone biosynthesis were more active in the high-risk group, whereas pathways involved in antigen processing and presentation, apoptosis, B cell receptor signaling, T cell receptor signaling, and JAK-STAT signaling were significantly inhibited (**Figure 8J**). These findings suggest that reduced immune cell infiltration in the high-risk tumor microenvironment may contribute to a poorer prognosis.

### 3.6 Predictive power of tumor risk score for immunotherapy benefit

**Figure 9:**
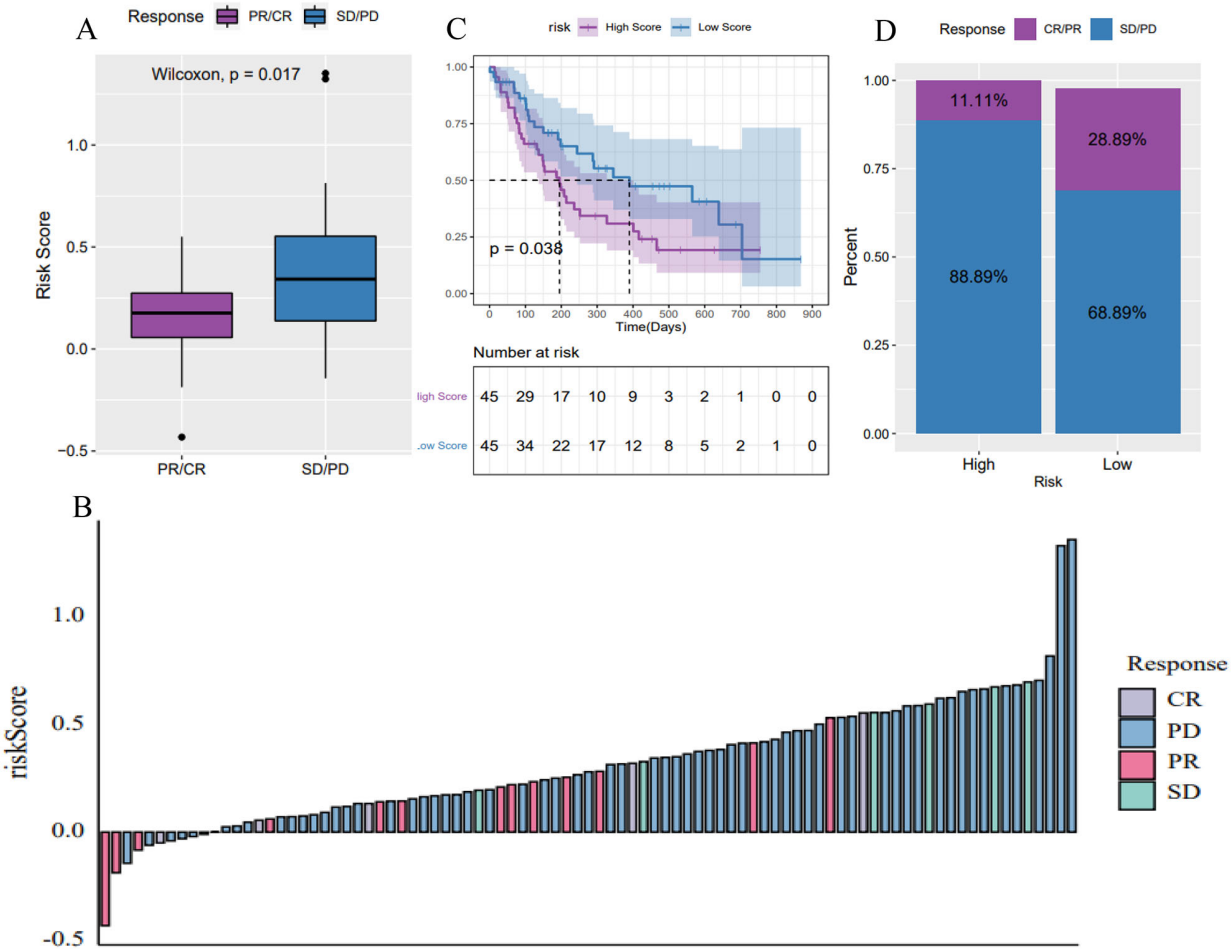
Predictive power of the 6-lncRNA risk score model for immunotherapy benefit in HNSC. **(A, B)** Risk scores of patients responding (CR/PR) versus non-responding (SD/PD) to immune checkpoint blockade (ICB) immunotherapy in the GSE176307 dataset. **(C)** Kaplan-Meier survival curves showing longer survival for low-risk patients receiving ICB. **(D)** Objective response rates to ICB, with higher rates in the low-risk group, suggesting the risk score’s predictive ability for immunotherapy benefit.

To evaluate the predictive capability of the tumor risk score for immunotherapy outcomes, we analyzed the GSE176307 dataset from the GEO database. Patients who underwent ICB immunotherapy were stratified into high and low-risk score groups. Notably, patients who responded to ICB, including those with a complete response (CR) and partial response (PR), had significantly lower risk scores compared to non-responders, including those with stable disease (SD) and progressive disease (PD) (**Figures 9A, B**). Patients in the low-risk score group had significantly longer survival (**Figure 9C**) and a higher objective response rate to ICB compared to the high-risk group (**Figure 9D**). These results suggest that the pyroptosis-related lncRNA risk score may be associated with patient’s response to immunotherapy.

### 3.7 Validation of lncRNA expression in the tumor risk score model

**Figure 10:**
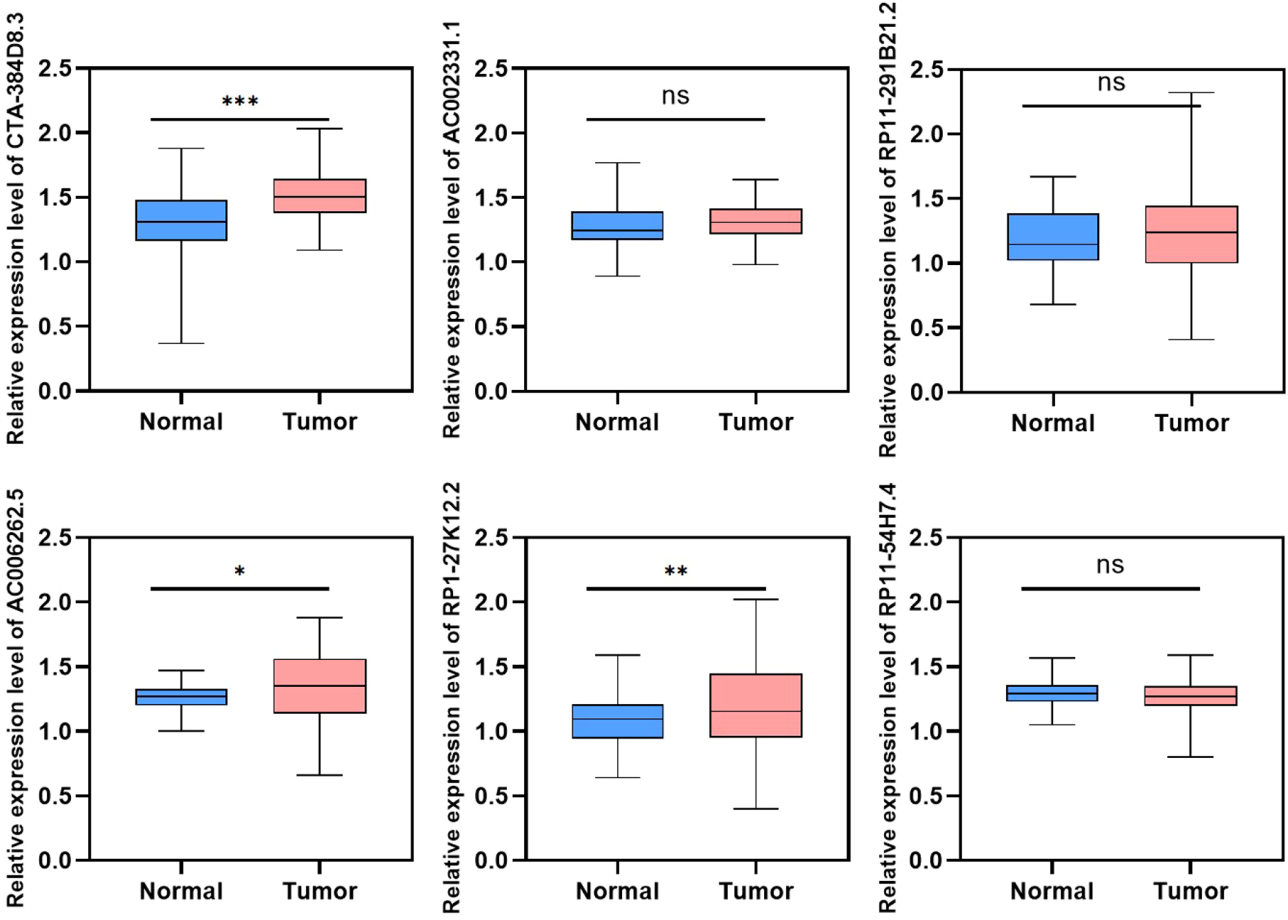
Validation of lncRNA expression in HNSC tumor and adjacent normal tissues using RT-qPCR. Relative expression levels of six lncRNAs (AC002331.1, CTA-384D8.35, RP11-291B21.2, AC006262.5, RP1-27K12.2, and RP11-54H7.4) were measured in HNSC tumor tissues and normal controls. Data are presented as mean ± SD. *p < 0.05, **p < 0.01, ***p < 0.001. ns refers to the absence of significant statistical significance.

To validate the expression of the 6 lncRNAs included in the tumor risk score model, we collected tumor and adjacent normal tissues from HNSC patients and performed reverse transcription quantitative PCR (RT-qPCR). The results, presented in **Figure 10** and **Supplementary Table S1**, demonstrated that AC006262.5, CTA-384D8.35, and RP1-27K12.2 were significantly upregulated in HNSC tumor tissues compared to normal controls. In contrast, AC002331.1, RP11-291B21.2, and RP11-54H7.4 showed no significant differences in expression between tumor and normal tissues. Interestingly, the relative expression level of CTA-384D8.35 was 1.31 ± 0.26 in the normal group and 1.51 ± 0.17 in the tumor group. The expression of this gene was significantly increased in the tumor group (t=-6.203, P<0.001).

## 4 Discussion

This study investigated the association between 18 pyroptosis-related genes and OS in HNSC patients using the TCGA_HNSC dataset. While most of these genes did not show a significant correlation with OS, high expressions of NAIP, NLRP1, and ZBP1 were notably linked to improved patient survival. Unsupervised clustering analysis identified two distinct pyroptosis subtypes, Cluster A and Cluster B, with Cluster A demonstrating a better median survival time compared to Cluster B. Differential gene expression analysis revealed 11 lncRNAs associated with these subtypes. Subsequently, a 6-lncRNA risk score model was developed using LASSO Cox regression. High-risk patients were found to have a worse prognosis, reduced immune cell infiltration, and higher tumor purity, indicating an immunosuppressive tumor microenvironment.

Additionally, the risk score model effectively predicted patient responses to ICB immunotherapy, with low-risk patients experiencing better therapeutic outcomes. These findings highlight the potential of pyroptosis-related lncRNAs as prognostic biomarkers and their utility in tailoring immunotherapy strategies for HNSC patients.

lncRNAs have been shown to play critical roles in regulating cell pyroptosis. For instance, Liang et al. demonstrated that knockdown of lncRNA MALAT1 induced pyroptosis in cervical cancer cells via the miR-124/SIRT1 regulatory axis (28). Similarly, Xu et al. found that lncRNA XIST facilitated pyroptosis in non-small cell lung cancer (NSCLC) cells through NLRP3, thereby enhancing chemosensitivity to cisplatin (29). However, the functional roles and mechanisms of lncRNAs in regulating pyroptosis in tumor cells, particularly in head and neck squamous cell carcinoma (HNSC), remain poorly understood. To address this gap, we utilized Pearson correlation analysis to identify pyroptosis-associated lncRNAs from gene expression data.

Our study utilized coexpression networks of pyroptosis-related genes and lncRNAs to construct a 6-lncRNA risk score model, including AC002331.1, CTA-384D8.35, RP11-291B21.2, AC006262.5, RP1-27K12.2, and RP11-54H7.4. Some of these lncRNAs have been previously implicated in tumor biology. For example, high expression of CTA-384D8.35 has been linked to better prognosis in triple-negative breast cancer and lung squamous cell carcinoma (29, 30). RP11-291B21.2 correlates with response to Durvalumab in NSCLC and bladder urothelial carcinoma (31). while AC006262.5 functions as an oncogene in hepatocellular carcinoma via the miR-7855-5p-BPY2C axis (32). Despite these insights, further exploration is needed to fully elucidate the role of pyroptosis-related lncRNAs in HNSC and their prognostic capabilities.

Given the pro-inflammatory nature of pyroptosis and its impact on the tumor immune microenvironment (16, 33, 34), we analyzed immune cell infiltration and immune-related pathways in the HNSC dataset. The high-risk group, defined by our 6-lncRNA risk score model, exhibited notably lower infiltration of key immune cells, such as CD4+ and CD8+ T cells. These cells are critical for anti-tumor immune responses, which may explain the better prognosis and response to immunotherapy observed in patients with lower tumor risk scores (35, 36). Interestingly, we also observed upregulation of regulatory T cells in the low-risk group, consistent with findings related to pyroptosis-associated gene signatures in tumors (34, 37). While regulatory T cells have traditionally been considered to suppress anti-tumor T cells and contribute to an immunosuppressed microenvironment, recent studies suggest that their role may vary across different tumors (38, 39). Moreover, distinct subtypes of regulatory T cells can have opposing effects on cancer prognosis (40).

The reduced infiltration of cytotoxic cells in the high-risk group suggests an “immunologically cold” tumor microenvironment, which is often associated with poorer responses to ICB therapies. Conversely, the low-risk group exhibited a higher degree of immune cell infiltration, indicative of an “immunologically hot” tumor microenvironment that may be more responsive to ICB therapy. Functional enrichment analysis supported these observations, showing upregulation of immunoregulatory pathways in the low-risk group, which are crucial for effective anti-tumor immune responses. The predictive capability of the 6-lncRNA risk score model was further validated in the GSE176307 dataset, where patients in the low-risk group not only had a higher objective response rate to ICB therapy but also demonstrated significantly longer survival compared to the high-risk group. These results highlight the potential of the 6-lncRNA risk score as a biomarker for selecting HNSC patients who are more likely to benefit from ICB therapy.

## 5 Conclusion

In this study, we investigated the association between pyroptosis-related genes and overall survival (OS) in patients with head and neck squamous cell carcinoma (HNSC) using the TCGA_HNSC dataset. Our analysis identified two distinct pyroptosis subtypes, with Cluster A demonstrating a better median survival time compared to Cluster B. Differential gene expression analysis revealed 11 lncRNAs associated with these subtypes, and further selection through LASSO Cox regression resulted in the development of a 6-lncRNA risk score model. The six lncRNAs included in this model are AC002331.1, CTA-384D8.35, RP11-291B21.2, AC006262.5, RP1-27K12.2, and RP11-54H7.4. High-risk patients, as classified by our model, exhibited a poorer prognosis and had a higher proportion of deaths compared to low-risk patients. The model was validated using an independent dataset, demonstrating its robustness and potential clinical utility. Additionally, we found that the risk score model effectively predicted patient responses to ICB immunotherapy, with low-risk patients experiencing better therapeutic outcomes. Our study provides new insights into the association between pyroptosis-related lncRNAs and HNSC prognosis, and establishes a prognostic model that could guide personalized immunotherapy strategies for HNSC patients.

## Data Availability

The raw data supporting the conclusions of this article will be made available by the authors, without undue reservation.

## Supplementary material

Supplementary Figure 1 The correlation between lncRNAs and pyroptosis-related genes. We combined a specific list of genes related to apoptosis and batch calculated the Spearman correlation coefficients and p-values between these genes and specified lncRNAs. Through correlation analysis, we found that these 6 apoptosis related lncRNAs were significantly correlated with 18 apoptosis related genes.

Supplementary Figure 2 Validation of the 6-lncRNA risk score model in the training and overall TCGA_HNSC data set. **(A)** Proportion of death samples in high and low-risk groups in the test set. **(B)** Kaplan-Meier survival curves in the test set showing poorer survival in the high-risk group. **(C)** ROC curves for the test set with 1-, 3-, and 5-year AUCs of 0.625, 0.610, and 0.671, respectively. **(D–F)** Risk score distribution, survival status and ROC curves of patients in overall TCGA_HNSC data set.

Supplementary Table 1 The primers used for RT-qPCR.
