# Supplementary figures and images for: A pyroptosis-related lncRNA risk model for the prediction of prognosis and immunotherapy response in head and neck squamous cell carcinoma
Source: Front Oncol. 2024 Nov 12;14:1478895. doi: 10.3389/fonc.2024.1478895 (PMC11588584; doi:10.3389/fonc.2024.1478895)

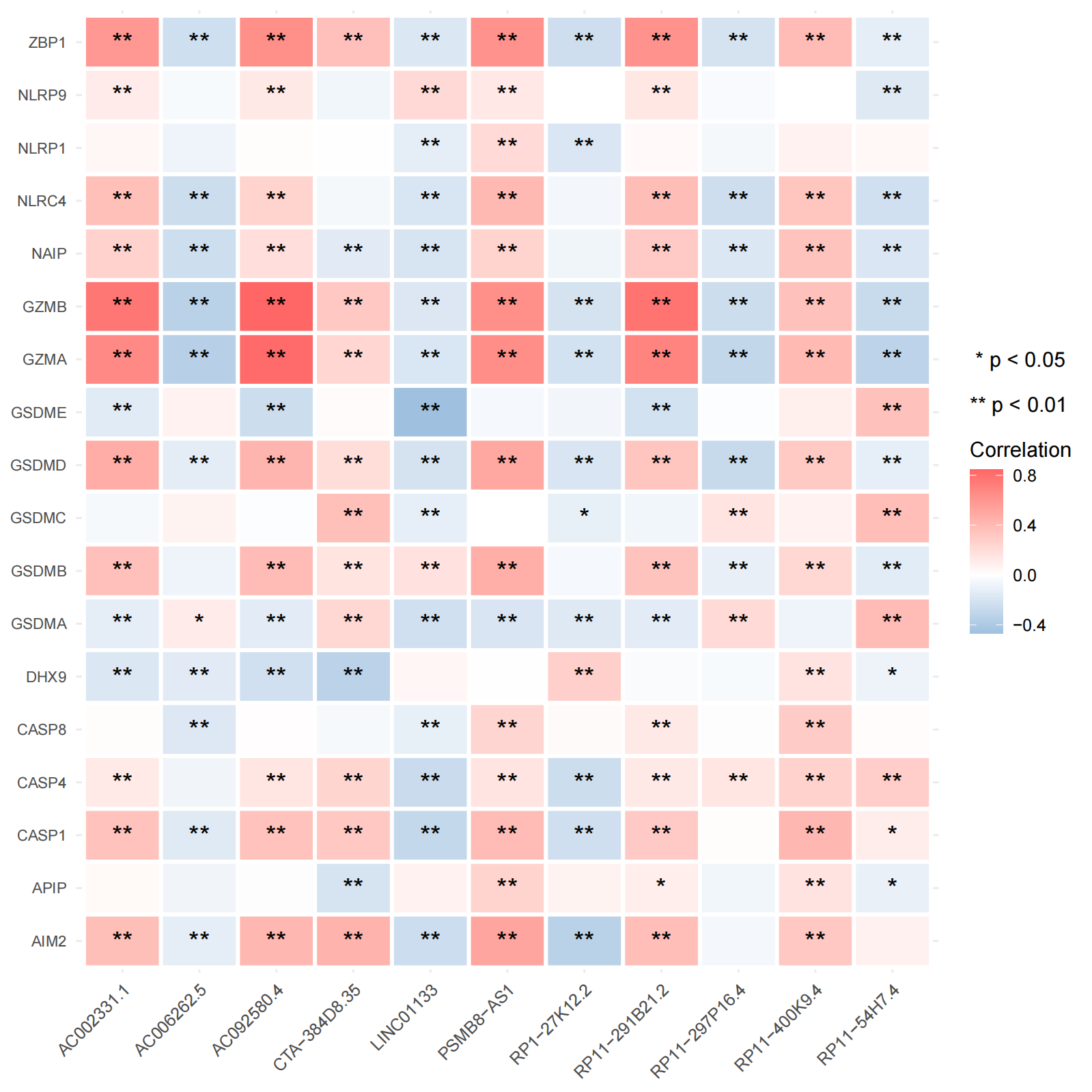

Supplement: Supplementary Figure 1 — The correlation between lncRNAs and pyroptosis-related genes. We combined a specific list of genes related to apoptosis and batch calculated the Spearman correlation coefficients and p-values between these genes and specified lncRNAs. Through correlation analysis, we found that these 6 apoptosis related lncRNAs were significantly correlated with 18 apoptosis related genes. [file Image1.tif]

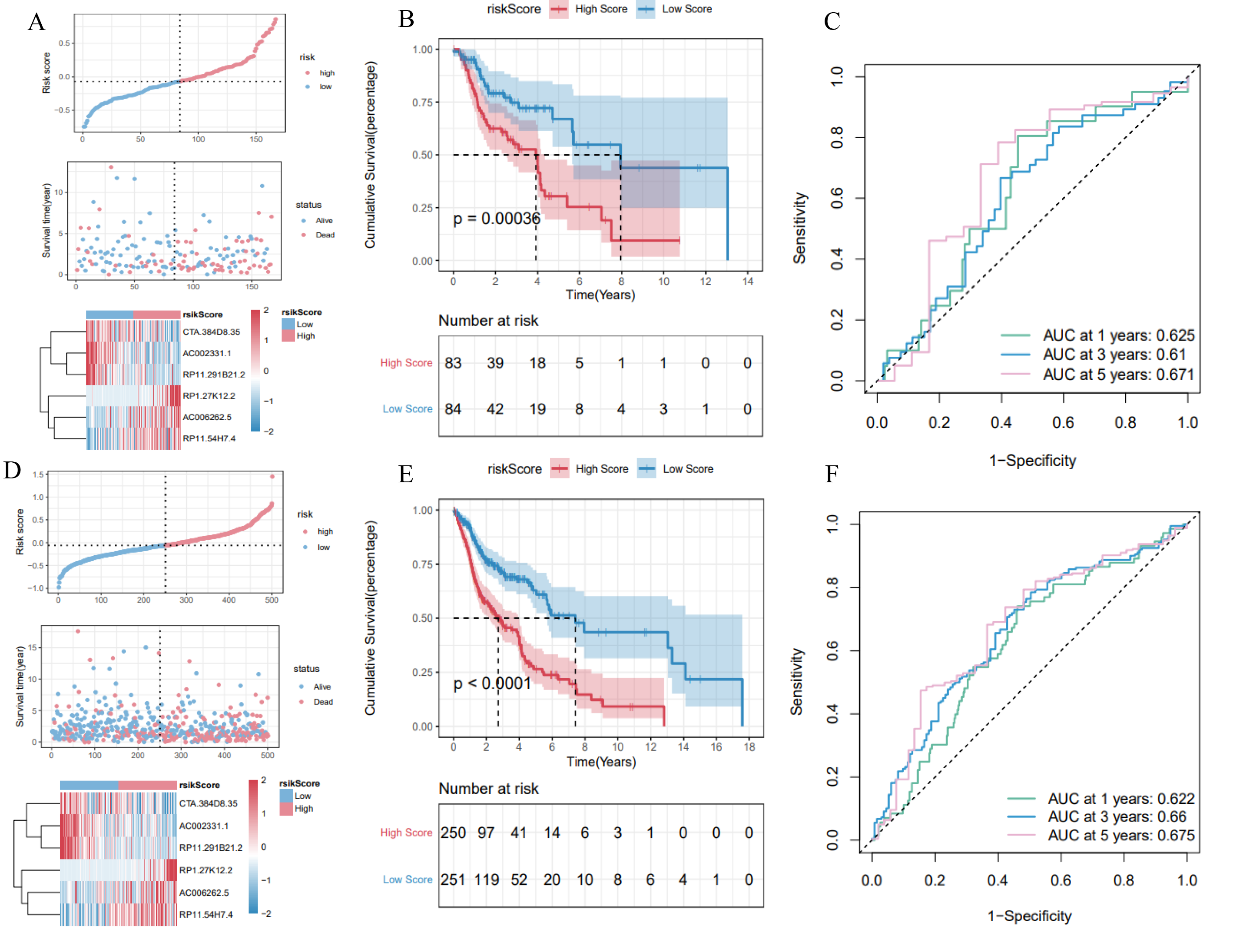

Supplement: Supplementary Figure 2 — Validation of the 6-lncRNA risk score model in the training and overall TCGA_HNSC data set. (A) Proportion of death samples in high and low-risk groups in the test set. (B) Kaplan-Meier survival curves in the test set showing poorer survival in the high-risk group. (C) ROC curves for the test set with 1-, 3-, and 5-year AUCs of 0.625, 0.610, and 0.671, respectively. (D–F) Risk score distribution, survival status and ROC curves of patients in overall TCGA_HNSC data set. [file Image2.tif]
