# Supplementary material for: A pyroptosis-related lncRNA risk model for the prediction of prognosis and immunotherapy response in head and neck squamous cell carcinoma
Source: Front Oncol. 2024 Nov 12;14:1478895. doi: 10.3389/fonc.2024.1478895 (PMC11588584; doi:10.3389/fonc.2024.1478895)
Supplement: Supplementary Table 1 — The primers used for RT-qPCR. [file Table1.docx]

Table S1 The primers used for RT-qPCR

| Genes | Forward | Reverse |
| --- | --- | --- |
| RP11-54H7.4 | 5’-CCGTCCTGTCTCCCTACTGA-3’; | 5’-AAAGTACAGGCCGTCATGGG-3’ |
| RP11-291B21.2 | 5’-CCCCCGAGGAGAAGATAGGAA-3 | 5’-TGACAGACGGTCCCTGCTTT-3’ |
| CTA-384D8.35-001 | 5’-AGGCGTCCTCATCCCTTTTC-3’ | 5’-GGCGATCCACGTCATTGAGA-3’ |
| AC002331.1 | 5’-CCACATACTCTGGGGAAACCT-3’ | 5’-GTGCTCCACACAGTCCTTCAG-3’ |
| AC006262.5 | 5’-GCCCAGATCAACAGAATCACG-3’ | 5’-ATGTGTCAGTTTGCTGGTGC-3’ |
| RP1-27K12.2 | 5’-AAGCATCCACGGCTAGTGTT-3’ | 5’-TGGGAGTGAGGAGAAGCAGT-3’ |
